# Supplementary material for: Refining the serum miR-371a-3p test for viable germ cell tumor detection
Source: Sci Rep. 2023 Jun 29;13:10558. doi: 10.1038/s41598-023-37271-1 (PMC10310745; doi:10.1038/s41598-023-37271-1)
Supplement: Supplementary file 1 — Supplementary Information. [file 41598_2023_37271_MOESM1_ESM.docx]

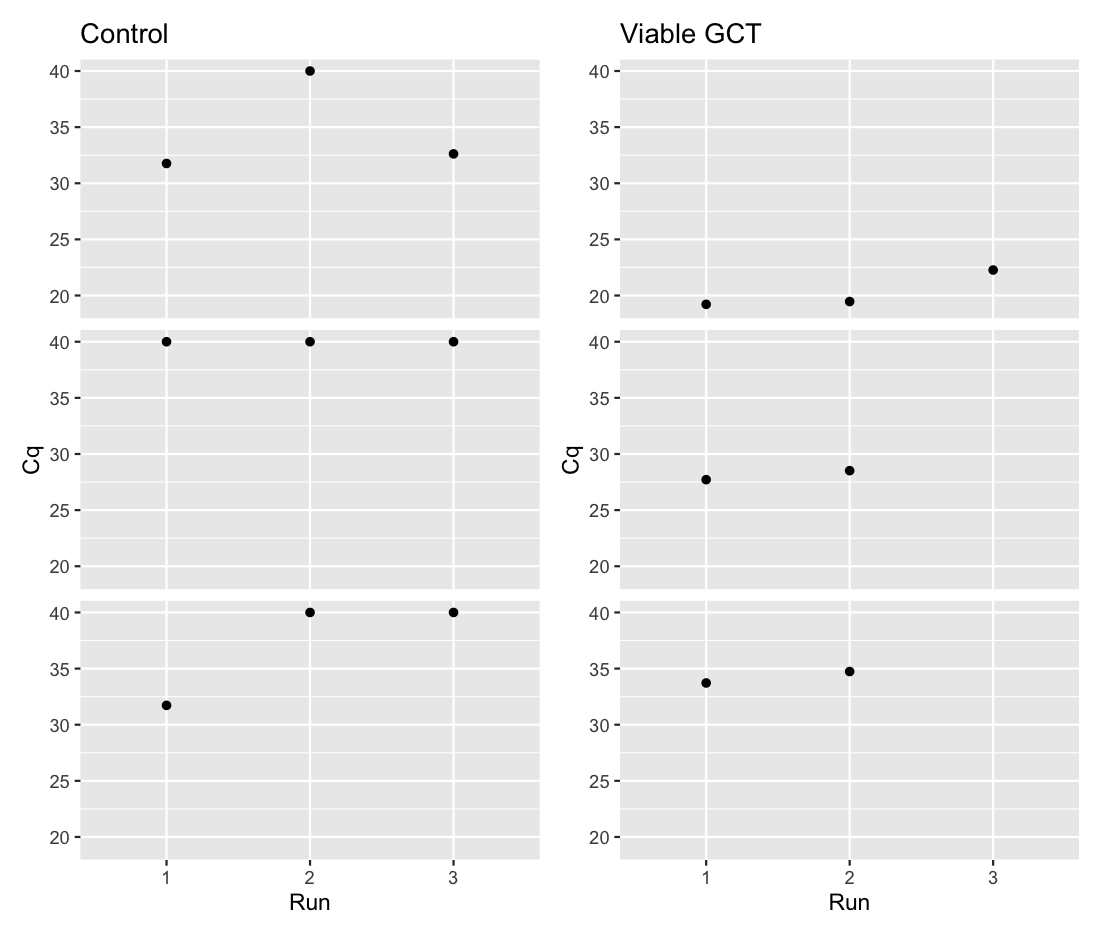


**Supplementary Figure 1. Examples of inconsistent results from the serum miR-371a-3p assay.** Representative data from multiple runs from 3 Control samples (left column) and 3 Viable GCT samples (right column). Each dot represents a mean Cq value of 3 replicate qPCR wells for a given sample. Each run per sample originated from the same RNA extract.


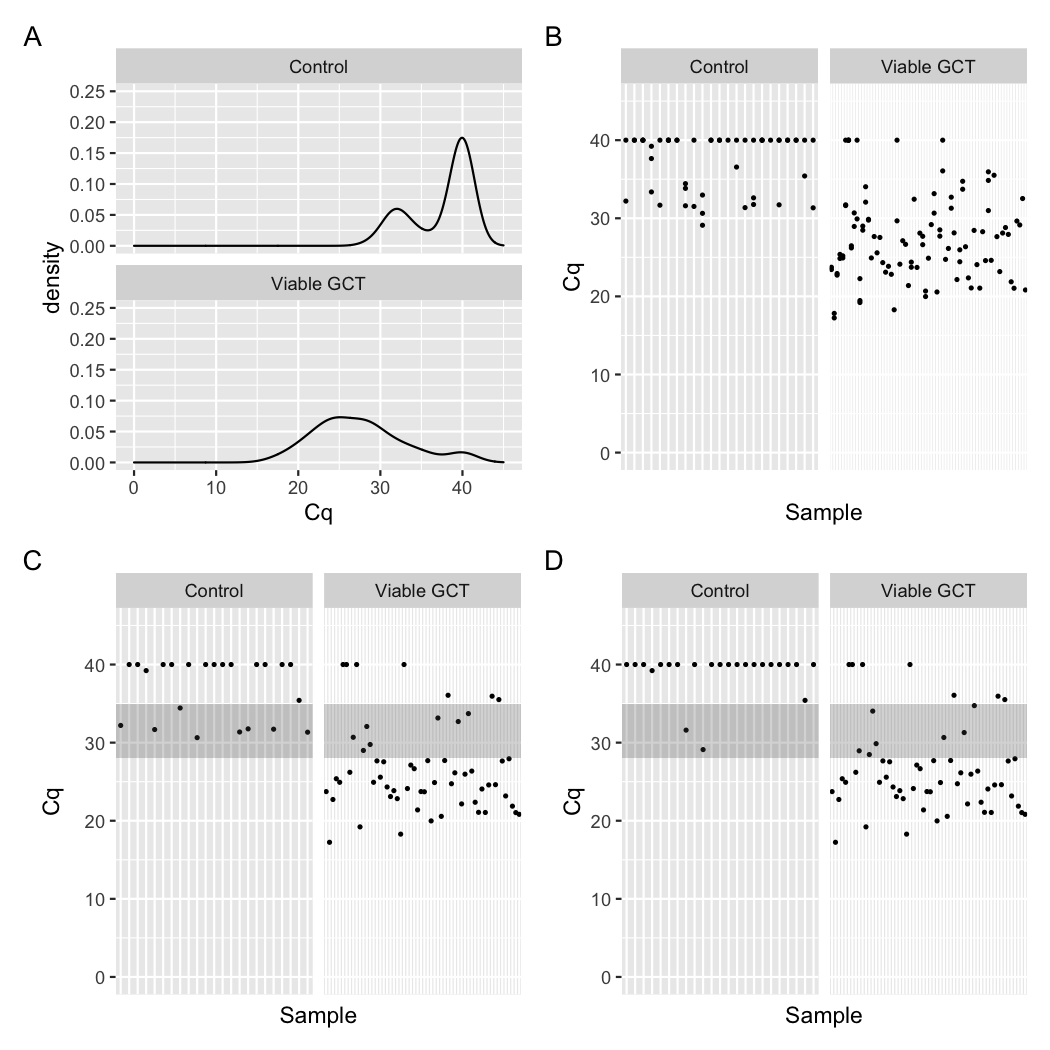


**Supplementary Figure 2. Additional data from aggregated 1° RPLND and orchiectomy patient cohorts. A:** Density plot of all data points from 1° RPLND and orchiectomy data sets. **B:** Individual Cq values of all data points from 1° RPLND and orchiectomy data sets. **C-D:** Cq values of serum miR-371a-3p using original (A) and revised (B) methodology (one point per sample).

| **Target** | **Supplier** | **Product no.** | **ID** |
| --- | --- | --- | --- |
| Cel-miR-39-3p | ThermoFisher | 4427975 | 000200 |
| Hsa-miR-30b-5p | ThermoFisher | 4427975 | 000602 |
| Hsa-miR-371a-3p | ThermoFisher | 4427975 | 002124 |

**Supplementary Table 1. List of TaqMan primers and probes used in this study.**

|  | **Original** | **Revised** |
| --- | --- | --- |
| **Threshold** | 30.2 | 37.6 |
| **Sensitivity** | 0.80 | 0.92 |
| **Specificity** | 1.00 | 0.95 |
| **AUC** | 0.909 (0.849-0.970) | 0.954 (0.912-0.996) |
| **PPV** | 1.00 | 0.98 |
| **NPV** | 0.66 | 0.83 |
| **Accuracy** | 0.85 | 0.93 |

**Supplementary Table 2. Performance characteristics of Original and Revised serum miR-371a-3p assay method in aggregated 1° RPLND and orchiectomy cohorts.**

|  | **Original** | **Revised** |
| --- | --- | --- |
| **Threshold** | 31.3 | 35 |
| **Sensitivity** | 0.89 | 0.92 |
| **Specificity** | 0.8 | 0.92 |
| **AUC** | 0.898 (0.793-1) | 0.934 (0.835-1) |
| **PPV** | 0.83 | 0.92 |
| **NPV** | 0.86 | 0.92 |
| **Accuracy** | 0.84 | 0.92 |

**Supplementary Table 3. Performance metrics of Original and Revised methodology of serum miR-371a-3p test in patients with minimal residual disease.**
